# Sestrin-2, a repressor of PDGFRβ signalling, promotes cigarette-smoke-induced pulmonary emphysema in mice and is upregulated in individuals with COPD

**DOI:** 10.1242/dmm.013482

**Published:** 2013-08-29

**Authors:** Juliana Heidler, Athanasios Fysikopoulos, Frank Wempe, Michael Seimetz, Thorsten Bangsow, Ana Tomasovic, Florian Veit, Susan Scheibe, Alexandra Pichl, Friederike Weisel, K. C. Kent Lloyd, Peter Jaksch, Walter Klepetko, Norbert Weissmann, Harald von Melchner

**Affiliations:** 1 Department of Molecular Haematology, Goethe University Medical School, D-60590 Frankfurt am Main, Germany; 2 Excellence Cluster Cardiopulmonary System (ECCPS), Justus-Liebig-University Giessen, Department of Internal Medicine, Universities of Giessen and Marburg Lung Centre (UGMLC), D-35392 Giessen, Germany; 3 Mouse Biology Program, University of California Davis, Davis, CA 95616, USA; 4 Department of Thoracic Surgery, University Hospital of Vienna, A-1090 Vienna, Austria

## Abstract

Chronic obstructive pulmonary disease (COPD) is a leading cause of morbidity and mortality worldwide. COPD is caused by chronic exposure to cigarette smoke and/or other environmental pollutants that are believed to induce reactive oxygen species (ROS) that gradually disrupt signalling pathways responsible for maintaining lung integrity. Here we identify the antioxidant protein sestrin-2 (SESN2) as a repressor of PDGFRβ signalling, and PDGFRβ signalling as an upstream regulator of alveolar maintenance programmes. In mice, the mutational inactivation of *Sesn2* prevents the development of cigarette-smoke-induced pulmonary emphysema by upregulating PDGFRβ expression via a selective accumulation of intracellular superoxide anions (O_2_^−^). We also show that *SESN2* is overexpressed and PDGFRβ downregulated in the emphysematous lungs of individuals with COPD and to a lesser extent in human lungs of habitual smokers without COPD, implicating a negative SESN2-PDGFRβ interrelationship in the pathogenesis of COPD. Taken together, our results imply that SESN2 could serve as both a biomarker and as a drug target in the clinical management of COPD.

## INTRODUCTION

Chronic obstructive pulmonary disease (COPD) is a global epidemic of major proportions that is predicted to become the third most common cause of death and fifth most frequent cause of chronic disability by 2030 (http://www.who.int/respiratory/copd/burden/en/). Cigarette smoking is a major risk factor, but several predisposing genetic factors have also been implicated in the pathogenesis of COPD (Hersh et al., 2006; Repapi et al., 2010).

A major component of COPD is pulmonary emphysema caused by a progressive destruction of alveolar walls with consequent loss of respiratory function. Although the mechanisms causing the emphysema are largely unknown, reactive oxygen species (ROS) induced by cigarette smoke and/or other environmental pollutants are thought to gradually disrupt signalling pathways responsible for maintaining lung integrity (Tuder and Petrache, 2012).

SESN2 belongs to a family of highly conserved antioxidant proteins with poorly understood functions. In mammalian cells, SESN2 is believed to reduce oxidative stress by rescuing the peroxidase activity of overoxidised peroxiredoxins (Budanov et al., 2004) and by activating the transcription factor NRF2 (nuclear factor erythroid 2-related factor 2) (Bae et al., 2013), which is a potent antioxidant gene inducer. However, independently of its antioxidant function, SESN2 inhibits mammalian target of rapamycin (mTOR) (Budanov and Karin, 2008), a prometabolic serine/threonine kinase that controls protein synthesis, cell growth, autophagy and cell death. The activation of the rapamycin-sensitive component of mTOR (mTORC1) has been associated with reduced pathology in experimental and human emphysemas (Weichhart et al., 2008; Wempe et al., 2010; Yoshida et al., 2010). Thus, SESN2 seems to simultaneously block ROS accumulation and mTOR signalling, which are believed to have opposite effects in the pathogenesis of COPD.

We and others have previously reported that mice with an inactivating mutation of the small splice variant of the latent transforming growth factor beta 4 gene (*Ltbp4S* KO) are born with alveolar septation defects that worsen with age (Dabovic et al., 2009; Sterner-Kock et al., 2002). By the age of 4–5 months, *Ltbp4S* KO lungs develop symptoms reminiscent of the centrilobular emphysema that is associated with late-stage COPD (Sterner-Kock et al., 2002). This phenotype is partially rescued by the inactivation of SESN2 in *Ltbp4S/Sesn2* double-knockout mice (Wempe et al., 2010). Based on this observation, we hypothesised that the *Sesn2* mutation would protect mice from developing emphysema after chronic exposure to cigarette smoke (an animal model that more closely mimics human COPD than does the *Ltbp4S* KO mouse), and that *Sesn2* expression might be altered in the lungs of individuals with COPD.

Here we show that the mutational inactivation of *Sesn2* protects mice against developing cigarette smoke-induced pulmonary emphysema. Moreover, we identify SESN2 as a repressor of PDGFRβ signalling, and PDGFRβ signalling as an upstream regulator of alveolar maintenance programmes. We further show that SESN2 is highly overexpressed and PDGFRβ downregulated in the emphysematous lungs of individuals with advanced COPD and to a lesser extent in the lungs of habitual smokers without COPD. Overall, our results imply that SESN2 could serve as both a biomarker and as a drug target in the clinical management of COPD.

**TRANSLATIONAL IMPACT**

**Clinical issue**

Chronic obstructive pulmonary disease (COPD), a disease caused by chronic exposure to cigarette smoke and/or other environmental pollutants, is a global epidemic that is predicted to become the third most common cause of death and fifth most frequent cause of chronic disability by 2030. Pulmonary emphysema, caused by progressive breakdown of alveolar walls, is a major feature of COPD. It is thought that reactive oxygen species (ROS) generated by exposure to cigarette smoke and other environmental pollutants could disrupt signalling pathways that promote lung integrity. It has previously been shown that inactivation of SESN2, a highly conserved antioxidant protein, partially rescues the disease phenotype in a mouse model of genetically determined pulmonary emphysema. Based on this finding, the authors hypothesised that SESN2 could have a role in the development of the more common, environmentally driven form of pulmonary emphysema.

**Results**

The authors show that mutational inactivation of *Sesn2* in mice prevents cigarette-smoke-induced pulmonary emphysema. They provide evidence that protection is mediated by upregulation of PDGFRβ, a signalling factor that promotes lung development and alveolar maintenance by stimulating secretion of keratinocyte growth factor and expression of elastin. Consistent with a negative SESN2-PDGFRβ interrelationship in the pathogenesis of COPD, the authors show that SESN2 is highly overexpressed whereas PDGFRβ is downregulated in the emphysematous lungs of individuals with advanced COPD. SESN2 overexpression was also observed to a lesser extent in the lungs of habitual smokers without COPD.

**Implications and future directions**

Using a robust mouse model for the disease, the authors uncover a role for SESN2 in the pathogenesis of COPD caused by chronic exposure to cigarette smoke and/or other environmental pollutants. They demonstrate that upregulation of PDGFRβ signalling decreases the lung’s susceptibility to injury inflicted by chronic exposure to tobacco smoke. Further evidence for the inverse relationship between PDGFRβ signalling and SESN2 is provided by their analysis of human lungs. The results strongly suggest that SESN2 could serve as both a biomarker and as a drug target in the clinical management of COPD. Because SESN2 is a druggable enzyme, future studies will aim to develop and validate inhalable small-molecule antagonists for the treatment of COPD.

## RESULTS

### *Sesn2* knockout mice are protected against cigarette-smoke-induced pulmonary emphysema

**Fig. 1.**
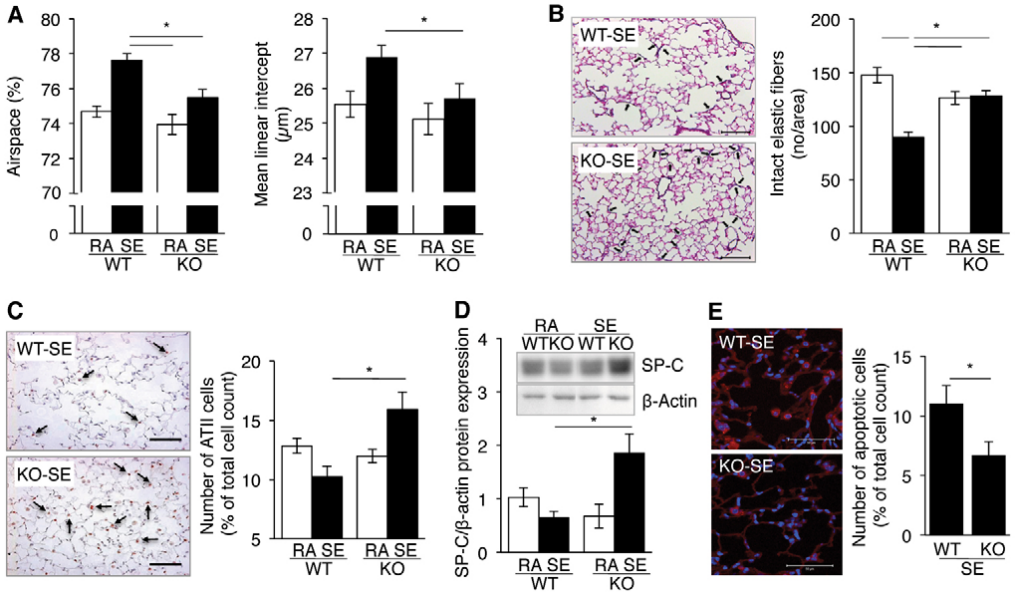
**Protection of *Sesn2* KO mice from cigarette-smoke-induced emphysema.** (A) Total airspace (left) and mean linear intercept (right) of lungs from wild type (WT) and *Sesn2* KO (KO) mice exposed to room air (RA) or cigarette smoke (smoke exposed; SE). Results are represented as means ± s.e.m. of *n*=7–14 individual mice per group. (B) Elastic fibres in lungs of WT and KO mice exposed to cigarette smoke. Left panel: representative histological sections stained for elastic fibres. Intact elastic fibres are marked by black arrows. Scale bars: 100 μm. Right panel: quantification of intact elastic fibres by light microscopy at 40× magnification. Elastic fibres were counted within an area of 0.07598346 mm^2^ using 175–370 images per section (see Materials and Methods for details). Results are represented as means ± s.e.m. of lungs from *n*=5 individual mice per group. (C) Reduction of alveolar type II (AVII) cells in mice exposed to cigarette smoke. Left panel: histological sections stained with an alveolar epithelial type II (ATII)-cell-specific TTF1 antibody. TTF1-positive cells (arrows) have brown stained nuclei. Scale bars: 100 μm. Right panel: frequency of TTF1-positive cells in WT and *Sesn2* KO mice exposed to room air (RA) or cigarette smoke (SE). The fraction of TTF1-positive cells was derived from a mean of 992±171 cells from three randomly chosen sections of each lung. Cells were counted at 20× magnification. Results are represented as means ± s.e.m. of lungs of *n*=4 individual mice per group. (D) Surfactant protein C (SP-C) expression by ATII cells in WT and KO mice exposed to room air (RA) or cigarette smoke (SE). Expression levels were estimated from western blots by densitometry (inlay: representative western blot). Results are represented as means ± s.e.m. of *n*=4 individual mice per group. (E) Frequency of apoptotic cells in lungs of WT and KO mice exposed to cigarette smoke. Left panel: confocal immunofluorescence microscopy of lung sections stained with anti-cleaved caspase 3 (CC3-Cy3) antibody. Apoptotic cells are red (Cy3) and nuclei blue (DAPI). Scale bars: 50 μm. Right panel: frequency of apoptotic cells obtained by counting 400±50 cells from six randomly chosen fields per lung. Results are represented as means ± s.e.m. of *n*=3 individual mice per group. *P<0.05.

Because the mutational inactivation of *Sesn2* in a genetic mouse model of pulmonary emphysema partially rescues the emphysema phenotype (Wempe et al., 2010), we wanted to investigate whether *Sesn2* inactivation would have a similar effect on the pathology of the medically more relevant, environmentally induced emphysemas. Towards this end, we exposed *Sesn2* knockout (KO) mice and corresponding wild-type (WT) controls to cigarette smoke for 8 months as previously described (Seimetz et al., 2011). During this time, the smoke-exposed WT mice developed pulmonary emphysema characterised by airspace enlargement, increased mean linear intercept (Fig. 1A) and a reduction of intact elastic fibres (Fig. 1B). Functionally, the mice exhibited an increased dynamic lung compliance and an elevated right ventricular systolic blood pressure (RVSP) (supplementary material Fig. S1). In contrast, the smoke-exposed *Sesn2* KO mice showed no statistically significant differences from non-exposed mice (Fig. 1A,B; supplementary material Fig. S1), suggesting that *Sesn2* inactivation protects against the development of emphysema.

Because cigarette smoke has been shown previously to induce apoptosis of alveolar epithelial type II (ATII) cells (Yoshida and Tuder, 2007), which are essential for lung structure maintenance, we quantified ATII cells in the lungs of non-exposed and smoke-exposed WT and *Sesn2* KO mice using thyroid transcription factor 1 (TTF1) as an ATII-cell-specific immunohistochemical marker (Kolla et al., 2007). Consistent with the previous studies, ATII cell numbers and expression of the ATII-cell-specific surfactant protein C (SP-C) were reduced in smoke-exposed WT mice as compared with non-exposed controls (Fig. 1C,D). In contrast, ATII cell numbers and SP-C expression were increased in smoke-exposed *Sesn2* KO mice, suggesting that these cells are more resistant against the toxic effects of cigarette smoke (Fig. 1C,D). Indeed, when apoptotic cells were quantified by cleaved caspase-3 (CC3) immunofluorescence staining, the smoke-exposed KO lungs contained fewer apoptotic cells as compared with the smoke-exposed WT lungs (Fig. 1E).

### *Sesn2* regulates PDGFRβ expression

Because earlier experiments involving Affymetrix Chip arrays suggested an upregulation of platelet derived growth factor receptor beta (PDGFRβ) in lung fibroblasts of *Sesn2* KO mice, and because PDGFR signalling is essential for lung morphogenesis and regeneration (Boström et al., 1996; Levéen et al., 1994), we hypothesised that PDGFRβ might participate in lung protection against emphysema caused by cigarette smoke. Therefore, we first tested whether PDGFRβ is upregulated in *Sesn2* KO lungs. Supplementary material Fig. S2A shows increased *Pdgfrβ* mRNA and protein levels in total lung tissue homogenates of *Sesn2* KO mice when compared with the corresponding control tissue. Similarly, staining of tissue sections with anti-PDGFRβ antibody revealed increased PDGFRβ expression in *Sesn2* KO lungs as compared with WT lungs.

**Fig. 2.**
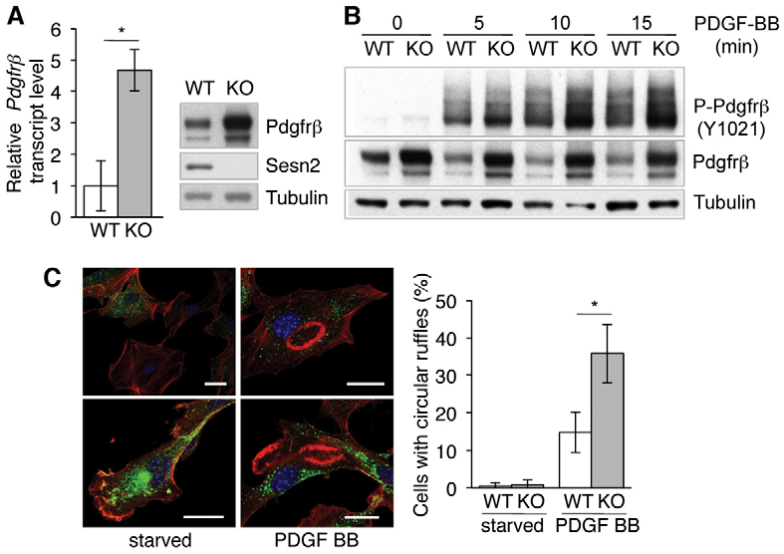
**Induction of *Pdgfrβ* expression in *Sesn2* KO-MLFs.** (A) Left panel: *Pdgfrβ* mRNA levels in WT and *Sesn2* KO (KO) MLFs quantified by qRT-PCR. Results are represented as means ± s.e.m. of *n*=4 independent experiments. Right panel: representative western blot. (B) Phosphorylated and total PDGFRβ protein levels in MLFs exposed to PDGF-BB. (C) Circular dorsal ruffle formation in MLFs exposed to PDGF-BB. Left panel: confocal images of circular dorsal ruffles. Ruffles, red (phalloidin); PDGFRβ, green (anti-PDGFRβ antibody); nuclei, blue (DAPI). Note the elevated levels of PDGFRβ in the KOMLFs. Scale bars: 20 μm. Right panel: quantitative assessment of circular dorsal ruffle formation. Differential counts were performed on a minimum of 100 cells per experiment. Results are represented as means ± s.e.m. of *n*=3 independent experiments. *P<0.05.

To investigate the SESN2-PDGFRβ interrelationship in more detail, we used mouse lung fibroblasts (MLFs) from WT (WTMLFs) and *Sesn2* KO (KO-MLFs) mice that were spontaneously immortalised and adapted for growth in tissue culture (Wempe et al., 2010). Both *Pdgfrβ* mRNA and protein were significantly upregulated in the KO-MLFs (Fig. 2A), resulting in a strong amplification of PDGFRβ signalling in response to the cognate platelet derived growth factor (PDGF-BB) ligand. Accordingly, PDGF-BB-stimulated KO-MLFs expressed significantly higher levels of phosphorylated PDGFRβ than the WT-MLFs (Fig. 2B), and exhibited increased membrane ruffling as a direct phenotypic consequence of PDGF stimulation (Fig. 2C) (Mellström et al., 1988).

To test whether PDGFRβ upregulation by *SESN2* inactivation could be replicated in human lung fibroblasts, we transduced embryonic lung-derived MRC5 cells and adult primary pulmonary fibroblasts with *SESN2*-shRNA-encoding lentiviruses. In both systems the knockdown of *SESN2* induced PDGFRβ expression (supplementary material Fig. S3).

**Fig. 3.**
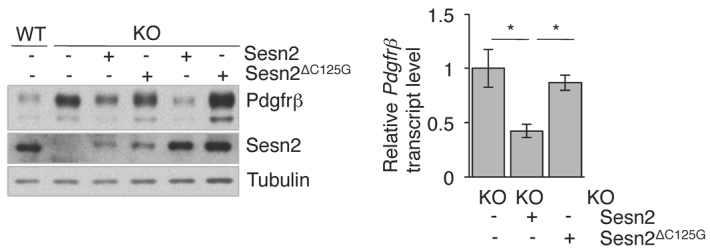
**Repression of *Pdgfrβ* expression by SESN2.** *Sesn2* KO-MLFs were transduced with lentiviral *Sesn2* cDNAs. *Pdgfrβ* expression was assayed in cell pools expressing comparable amounts of SESN2 and SESN2^ΔC125G^. Left panel: western blot showing PDGFRβ and lentiviral SESN2 expression in transduced KO-MLF cell pools. Right panel: *Pdgfrβ* mRNA levels quantified by qRT-PCR. Results are represented as means ± s.e.m. of *n*=3 separate experiments. *P<0.05.

Overall, the data suggest that SESN2 is a PDGFRβ repressor and, to test this directly, we re-expressed exogenous SESN2 in KO-MLFs. Fig. 3 shows that exogenous SESN2 downregulated *Pdgfrβ* mRNA and protein expression in a dose-dependent manner. However, downregulation was not observed with equivalent amounts of a catalytically inactive mutant (SESN2^ΔC125G^) (Budanov et al., 2004), indicating that the catalytic domain is required for *Pdgfrβ* repression.

### PDGFRβ activates alveolar maintenance programmes

**Fig. 4.**
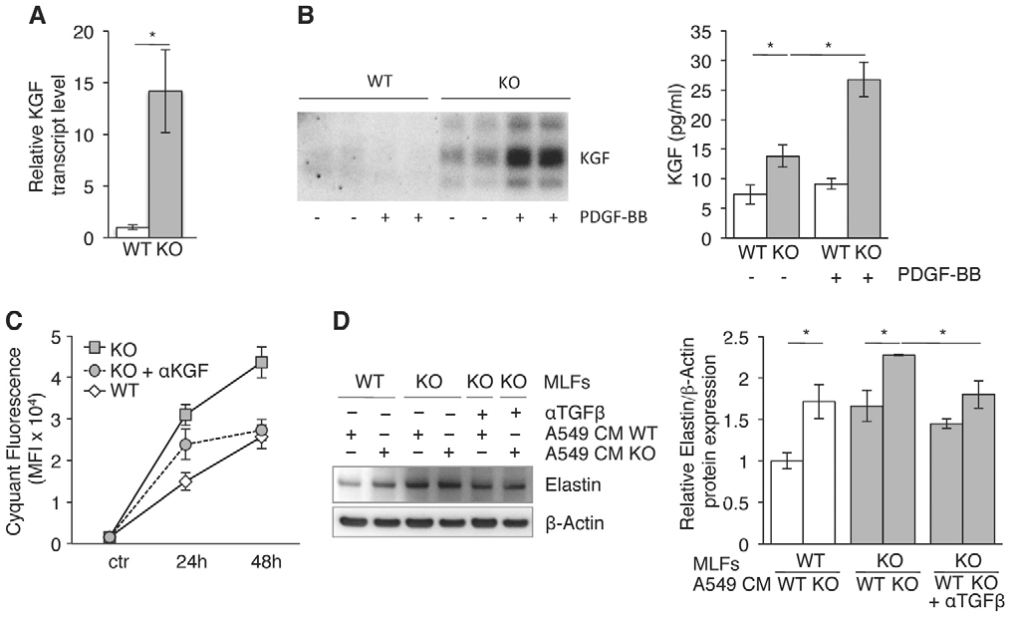
**Upregulation of alveolar maintenance programmes by SESN2 inactivation.** (A) *KGF* mRNA expression in WT and *Sesn2* KO-MLFs quantified by qRTPCR. (B) Western blot of tenfold concentrated CM (left) and KGF protein concentrations in straight CM measured by ELISA (right) prepared from MLFs before and after PDGF-BB stimulation. (C) A549 cell proliferation in the presence of straight MLF CM ± neutralising anti-KGF antibody estimated by the CyQuant proliferation test. (D) Elastin expression in WT- and KO-MLFs target cells incubated with doubly conditioned A549/WT-MLF and A549/KO-MLF media. Representative western blot (left) and elastin levels in MLFs (right) quantified by densitometry. All results are represented as means ± s.e.m. from *n*=3 separate experiments. *P<0.05.

Early studies on mesenchymal-epithelial cell interactions identified PDGF-BB as an inducer of keratinocyte growth factor [*KGF*; also known as fibroblast growth factor 7 (*FGF7*)] mRNA expression in human fibroblasts (Chedid et al., 1994). Because KGF is a well-known lung protective cytokine that stimulates ATII cell proliferation (Ware and Matthay, 2002) and ATII cells were directly affected by cigarette smoke in the smoking rodent model, we tested whether KGF is upregulated in KO-MLFs as a result of PDGFRβ signal amplification. As shown in Fig. 4A, *KGF* mRNA expression was upregulated over tenfold in KO-MLFs, leading to cytokine release into the culture medium so that conditioned medium (CM) of KO-MLFs contained significantly higher levels of KGF than did the CM of the WT-MLFs (Fig. 4B). Moreover, KGF secretion by KO-MLFs was induced even further by PDGF-BB, indicating that PDGFRβ is an upstream regulator of KGF expression (Fig. 4B).

To determine whether the secreted KGF was biologically active, we incubated ATII-cell-derived human A459 adenocarcinoma cells known to express the KGF receptor (Fischer et al., 2008) with WT-and KO-MLF CM. Fig. 4C shows that the KO-MLF CM, but not the WT-MLF CM, stimulated A549 proliferation under serum-free conditions. This effect was reversed by the addition of neutralising anti-KGF antibody, proving that the observed proliferation is mediated by KGF.

Previous observations have shown that KGF-pre-treated ATII cells upregulate the expression of elastin in co-cultured pulmonary fibroblasts, an activity that has been attributed to TGFβ1 (Yildirim et al., 2010). To test whether the KGF-containing KO-MLF CM would have a similar effect after preincubation with ATII cells, we incubated A549 cells with WT- or KO-MLF CM and added the doubly MLF/A549 CM back to WT- and KO-MLFs. Fig. 4D shows that the KO-MLF/A549 CM induced the expression of elastin in both WT- and KO-MLFs, whereas the equivalent WTMLF/A549 CM had no effect. This upregulation of elastin was partially blocked by neutralising pan-TGFβ antibody (Fig. 4D), suggesting that the elastin induction involves TGFβ. Like PDGFRβ, KGF and elastin expression were also elevated in *Sesn2* KO lungs when compared with normal lungs (supplementary material Fig. S2B,C).

Finally, to test whether PDGFRβ signalling has an impact on the mTOR pathway, we exposed WT- and KO-MLFs to PDGF-BB and estimated PDGFR and mTOR signalling by western blotting using antibodies against phosphorylated AKT (p-AKT) and P70S6K (p-P70S6K), respectively. Consistent with the downstream position of mTORC1 within receptor tyrosine kinase pathways (Manning, 2004), PDGF-BB induced the phosphorylation of P70S6K in both WT- and KO-MLFs – an effect that could be blocked by rapamycin (supplementary material Fig. S4).

Thus, the inactivation of *Sesn2* unleashes a sequence of lung protective mechanisms starting with PDGFRβ and concluding with elastin (supplementary material Fig. S5). This reduces the pulmonary susceptibility to emphysema induced by tobacco smoke, which upregulates *Sesn2* expression (supplementary material Fig. S6A) and thus suppresses the alveolar maintenance programmes controlled by PDGFRβ (supplementary material Fig. S6B–D).

**Fig. 5.**
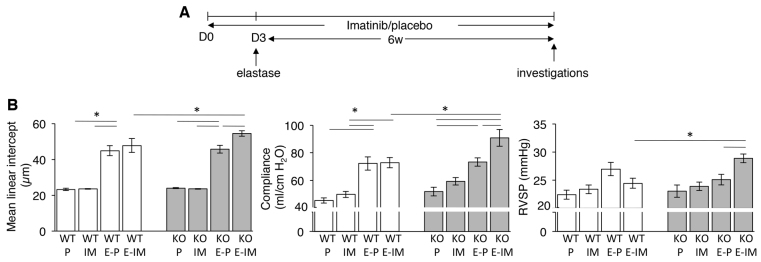
**Effect of the PDGFRβ antagonist imatinib on emphysema development in the mouse elastase emphysema model.** (A) Treatment scheme. Mice received placebo or imatinib once per day by oral gavage from day 0 (D0) onwards until week 6. Pancreatic elastase was administered intratracheally on day 3 (D3). (B) Mean linear intercept (left panel), dynamic lung compliance (middle panel) and right ventricular systolic blood pressure (RVSP) (right panel) of WT and *Sesn2* KO (KO) mice treated with elastase ± imatinib. P, placebo; IM, imatinib; E-P, elastase plus placebo; E-IM, elastase plus imatinib. Results are represented as means ± s.e.m. of *n*=5–7 individual mice per group. *P<0.05.

**Fig. 6.**
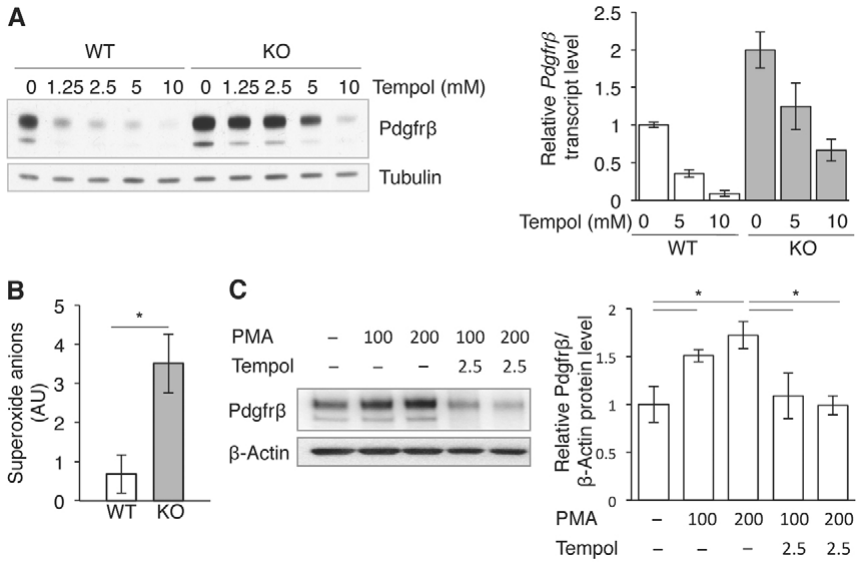
**Regulation of *Pdgfrβ* expression by superoxide anions.** (A) PDGFRβ protein (left) and mRNA (right) expression in MLFs exposed to tempol. Relative *Pdgfrβ* transcript levels are represented as means ± s.e.m. of three separate experiments. (B) Superoxide anion levels in MLFs quantified by electron paramagnetic resonance spectroscopy (EPR). Results are represented as means ± s.e.m. of six independent measurements. AU, arbitrary units. (C) PDGFRβ expression in MLFs exposed to phorbol 12-myristate 13-acetate (PMA) ± tempol. Left panel: representative western blot. Right panel: PDGFRβ levels quantified by densitometry. Results are represented as means ± s.e.m. of three separate experiments. *P<0.05.

### Inhibition of PDGFRβ signalling increases susceptibility to emphysema

To investigate whether PDGFRβ is important in the overall pathogenesis of emphysema, we employed the smoke-independent elastase-induced emphysema model (Shapiro, 2000). Groups of mice with elastase-induced emphysema were treated either with placebo or with the PDGFRβ inhibitor imatinib (Fig. 5A). Whereas imatinib had no effect on elastase-induced emphysema in WT mice, it significantly exacerbated the post-elastase lesions in the *Sesn2* KO lungs, suggesting that PDGFRβ signalling directly contributes to lung protection in these mice (Fig. 5B). The inability of imatinib to affect the outcome in post-elastase WT mice is most likely due to the steady-state PDGFRβ signalling, which is less susceptible to inhibition by imatinib (Hägerstrand et al., 2006).

### PDGFRβ expression requires superoxide anions (O_2_^−^)

Because KO-MLFs accumulate ROS (Budanov et al., 2002; Wempe et al., 2010) and ROS have been shown to stimulate PDGFRβ signalling (Choi et al., 2005), we next investigated whether the PDGFRβ activation by the *Sesn2* mutation is ROS dependent. To this end, we first exposed the MLFs to PEG-catalase, which neutralises hydrogen peroxide (H_2_O_2_). Supplementary material Fig. S7A shows that PEG-catalase had no significant effect on PDGFRβ expression, suggesting that H_2_O_2_ is not directly involved in this interaction. Similarly, exposure to H_2_O_2_ had only a slight effect on PDGFRβ in KO-MLFs although it strongly suppressed expression in the WT-MLFs, presumably by upregulating *Sesn2* (supplementary material Fig. S7B). However, exposure to the superoxide dismutase mimetic tempol significantly reduced *Pdgfrβ* mRNA and protein in both WT- and KO-MLFs (Fig. 6A), suggesting that the superoxide anions (O_2_^−^) are among the PDGFRβ-stimulating ROS subspecies. Indeed, when O_2_^−^ levels were measured directly by electron paramagnetic resonance spectroscopy (EPR), the KO-MLFs exhibited about fourfold higher levels than the WT-MLFs (Fig. 6B).

**Fig. 7.**
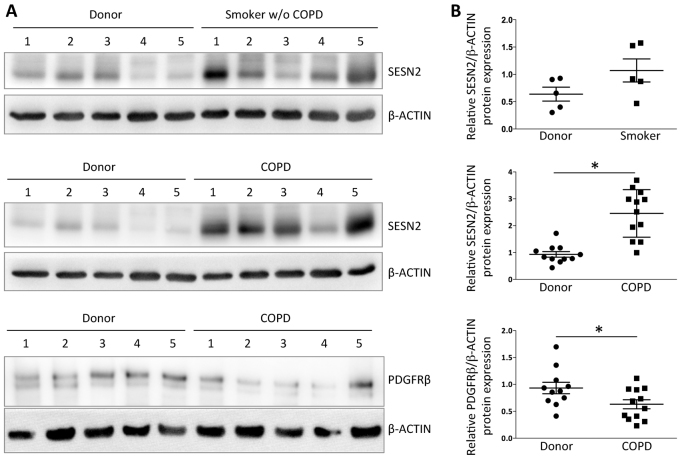
**SESN2 expression in lungs from healthy donors, habitual smokers without COPD and individuals with late-stage COPD.** (A) Representative western blots of five individuals per group. (B) SESN2 protein levels quantified by densitometry. Each entry corresponds to one individual (for patient characteristics see supplementary material Table S1). Mean values ± s.e.m. are indicated by straight lines. Healthy donor (*n*=11); smoker without COPD (*n*=5); individual with advanced COPD (*n*=12).

To directly test whether excess O_2_^−^ stimulates PDGFRβ expression, we exposed WT-MLFs to phorbol 12-myristate 13-acetate (PMA), which activates NADPH oxidase O_2_^−^ production. As expected, PMA upregulated PDGFRβ expression in a dose-dependent manner, an effect that was readily reversed by tempol (Fig. 6C).

Although we previously showed that peroxynitrite generated by an O_2_^−^–nitric-oxide (NO) interaction in the pulmonary vasculature promotes the development of emphysema (Seimetz et al., 2011), our present results indicate that O_2_^−^ is required for alveolar maintenance. Because *in situ* hybridisation experiments showed that *Sesn2* mRNA expression was confined to the subepithelial bronchial space, where it colocalised in part with smooth muscle actin (α-SMA), suggesting expression by myofibroblasts (supplementary material Fig. S8), the beneficial and deleterious effects of superoxide anions are most likely cell-type specific.

### *SESN2* is upregulated in lungs of individuals with COPD

We assessed *SESN2* expression in human lung samples from healthy donors, individuals with advanced COPD and habitual smokers without COPD. We found significantly upregulated *SESN2* expression in lungs of habitual smokers and especially in lungs of individuals with advanced COPD when compared with healthy donor lungs (Fig. 7; supplementary material Table S1). Moreover, PDGFRβ levels were reduced significantly in COPD lungs when compared with normal lungs, strongly supporting a negative SESN2-PDGFRβ interrelationship as a mechanism in the pathogenesis of COPD.

## DISCUSSION

Our findings indicate that SESN2 has a crucial role in the pathogenesis of pulmonary emphysema. Its upregulation in mice exposed to cigarette smoke reduced alveolar maintenance, as reflected by the reduction of ATII cells, increased apoptosis and increased fragmentation of elastic fibres. These toxic effects of cigarette smoke were prevented by the mutational inactivation of *Sesn2*, which protected *Sesn2* KO mice from the development of emphysema. These results substantiate our previous observations showing improved lung pathology in a genetic mouse model of COPD carrying the *Sesn2* mutation (Wempe et al., 2010).

In lung fibroblasts of mouse and human origin, the inactivation of SESN2 induced PDGFRβ expression and amplified PDGFRβ signalling in response to ligand. This induced the expression KGF and elastin *in vitro* and *in vivo*, suggesting that PDGFRβ is a master regulator of lung remodelling and injury repair (supplementary material Fig. S5). Consistent with this interpretation, the inhibition of PDGFRβ signalling in *Sesn2* KO mice by imatinib significantly exacerbated the pulmonary lesions induced by the local application of elastase. In this regard, it might be relevant that patients treated with imatinib for chronic myeloid leukaemia were reported to develop interstitial lung disease with fibrosis as a therapeutic side effect (Ohnishi et al., 2006).

We and others have shown that SESN2 inactivation leads to ROS accumulation and oxidative stress (Budanov et al., 2004; Wempe et al., 2010), which is believed to play a role in the pathogenesis of a variety of diseases, including COPD (Tuder and Petrache, 2012). However, ROS have also been identified as crucial regulators of a variety of signal transduction pathways (Finkel, 2011). PDGFRβ signalling, for example, is induced by ROS accumulating in mouse embryonic fibroblasts lacking the ROS scavenger peroxiredoxin II via a ROS-mediated inhibition of PDGFRβ inactivating phosphatases (Choi et al., 2005). Because, in some cellular contexts, SESN2 is responsible for maintaining peroxiredoxin function (Budanov et al., 2004), a similar effect might be expected in cells lacking SESN2. However, two considerations suggest that a disabled peroxiredoxin rescue mechanism is unlikely in the MLFs. First, by inducing PDGFRβ protein and mRNA expression, SESN2 does not directly interfere with PDGFRβ signalling, as one would expect from a SESN2-peroxiredoxin interaction. Second – and consistent with several other studies (Thamsen et al., 2011; Woo et al., 2009) – we were unable to demonstrate peroxiredoxin reductase activity for SESN2 in the pulmonary fibroblasts (data not shown).

Quantification of ROS by EPR revealed highly increased superoxide (O_2_^−^) levels in *Sesn2* KO-MLFs. Because the selective manipulation of these levels by tempol and PMA had a direct impact on PDGFRβ expression, we conclude that the O_2_^−^ radicals are among the PDGFRβ-regulating ROS subspecies. Thus, by lowering intracellular O_2_^−^, SESN2 either directly or indirectly inhibits PDGFRβ expression.

Another mechanism possibly involved in PDGFRβ repression relates to the recently reported ability of SESN2 to activate NRF2, which is a global transcriptional activator of antioxidant gene expression, including *SESN2* (Bae et al., 2013; Shin et al., 2012). NRF2-induced antioxidant activity could therefore reduce the superoxide anions required for PDGFRβ expression. Although this mechanism is presently under investigation, it is important to note that, unlike the PDGFRβ repression described here, SESN2 does not seem to require its catalytic function for activating NRF2 (Bae et al., 2013).

It is believed that ROS induced by cigarette smoke and other environmental pollutants gradually disrupt signalling pathways responsible for maintaining lung integrity (Tuder and Petrache, 2012; Yoshida et al., 2010). However, our present results clearly show that ROS are also required for the activation of these pathways, which might explain why COPD treatment with antioxidants has been unsuccessful (Rahman, 2012; Rahman and MacNee, 2012).

We conclude that SESN2 has a negative effect on the development of pulmonary emphysema by suppressing signal transduction pathways that are important for lung injury repair. Its consistent upregulation in the emphysematous lungs of individuals with advanced COPD suggests that they might benefit from treatment with antagonists of SESN2 function. Moreover, because SESN2 levels were also higher in human lungs of habitual smokers without COPD, long before the onset of deteriorated lung structure and function, the protein could also serve as a potential biomarker in the clinical management of COPD.

## MATERIALS AND METHODS

### Reagents

Antibodies against the following proteins were used: SESN2 (#10795-1-AP, Proteintech Group), TTF1 (#MS-699-PO, Thermo Scientific), CC3 (#9661, Cell Signaling Technology), PDGFRβ (#4564, Cell Signaling Technology), pPDGFRβ-Y1021 (#2227, Cell Signaling Technology), KGF (AF-251-NA, R&D Systems), elastin (#PR385, Elastin Products Company), panTGFβ (AB-100-NA, R&D Systems), pan-Akt (#4601, Cell Signaling Technology), pAKT-S473 (#4060, Cell Signaling Technology), P70S6K (#2708, Cell Signaling Technology), pP70S6K-T389 (#9234, Cell Signaling Technology), β-actin (A2228, Sigma), tubulin (#2125, Cell Signaling Technology). Cytokines used were: hPDGF-BB (Sigma). Other reagents were: H_2_O_2_ (Sigma), tempol (R&D Systems), PEG-catalase (Sigma), PMA (Sigma), porcine pancreatic elastase (PPE: ET947, Elastin Products Company), rapamycin, Imatinib (*Glivec*^®^, Novartis).

### Mice

All experiments were approved by the governmental ethics committee for animal welfare (Regierungspräsidium Giessen, Germany). Experiments were performed with 8- to 10-week-old C57BL6/J WT and C57BL6/J *Sesn2* KO mice [RRJ141/*Sesn2^Gt(RRJ141)Byg^*]. RRJ141 (C57BL6/J/129P2) mice were rederived at UC-Davis (USA) from frozen embryos distributed by the UC-Davis branch of the Mutant Mouse Regional Resource Centres (MMRRCs) and backcrossed to C57BL6/J mice for at least ten generations before use. Genotyping was performed by genomic PCR using mouse tail DNA and primers complementary to sequences flanking the gene trap insertion site. All primers are available on request.

### Cigarette smoke exposure

WT and *Sesn2* KO mice were exposed to mainstream smoke of 3R4F cigarettes (Lexington, KY) at 140 mg particulate matter/m^3^ for 6 hours/day, 5 days/week for up to 8 months as previously described (Seimetz et al., 2011). Age-matched WT and *Sesn2* KO mice were kept under identical conditions without smoke.

### Elastase emphysema model and treatment with imatinib

Mice were anaesthetised prior to elastase treatment using a mixture of 5% isoflurane (Baxter) in O_2_. Anaesthetised mice suspended by their upper incisors received 100 μl of saline solution with or without 24 U/kg body weight (BW) porcine pancreatic elastase through an intratracheally inserted 20G × 1^1/4^ (1.1×33 mm) catheter tube (Vasofix^®^ Safety, B. Braun). Imatinib mesylate (100 mg/kg BW) or placebo were applied daily for 6 consecutive weeks by oral gavage using 200 μl of an aqueous solution.

### Lung fixation and alveolar morphometry

For alveolar morphometry, lungs were fixed with 4.5% paraformaldehyde in phosphate-buffered saline (pH 7.0) via the trachea at a pressure of 22 cm H_2_O. The mean linear intercept and air-space were measured after staining with haematoxylin and eosin (H&E). Total scans from each lung lobe were analysed using the Qwin software (Leica, Wetzlar, Germany) as previously described (Wempe et al., 2010).

### Lung compliance tests and *in vivo* haemodynamics

Mice were anaesthetised with ketamine (60 mg/kg BW) and xylazine (10 mg/kg BW) intraperitoneally and anticoagulated with heparin (1000 U/kg). The trachea was cannulated and the lungs were ventilated with room air (10 μl/g BW) at a rate of 150 breaths per minute using a positive end-expiratory pressure of 2.0 cm H_2_O. A tracheal cannula was connected to a pneumotachometer (Hugo Sachs Electronics, March-Hugstetten, Germany) and the dynamic compliance of the living animal was evaluated using the HSE PULMODYN software (Hugo Sachs Electronics, March-Hugstetten, Germany). Right ventricular systolic pressure was measured by inserting a PE-80 tube into the right ventricle via the right jugular vein as described previously (Dumitrascu et al., 2006).

### Cell cultures and lentiviral transductions

MLFs, A549 cells (ATCC, CCL-185) and MRC5 cells (ATCC, CCL-171) were grown in Dulbecco’s modified Eagle’s medium (DMEM, High Glucose, Sigma) supplemented with 10% fetal calf serum (FCS), 2 mM glutamine (Gibco) and 1% penicillin/streptomycin (Gibco). Primary human adult lung fibroblasts were purchased from PromoCell and grown according to the manufacturer’s instructions.

Lentiviral transductions of *Sesn2*, *Sesn2*^Δ^*^C125G^* (kindly provided by Peter Chumakov, Lerner Research Institute, Cleveland, OH), and of *Sesn2* and *luciferase* shRNAs (kindly provided by Elena Feinstein, Quark Pharmaceuticals, Ness Ziona, Israel) were performed with vesicular stomatitis virus G (VSVG) pseudotyped lentiviral particles at multiplicities of infections (MOIs) between 2 and 5. Briefly, cells were exposed to 15× concentrated supernatants collected over 24 hours from HEK 293T cells transfected with lentiviral and corresponding helper plasmids. After incubating for 16 hours, the supernatants were replaced with fresh medium. For the *Sesn2* knockdown (KD) experiments, cells were cultured for 5-8 days. For the *Sesn2/Sesn2*^Δ^*^C125G^* reconstitution experiments, stable transformants were isolated by limiting dilution and SESN2 expression was assessed by western blotting. Equal aliquots of clones expressing comparable SESN2 or SESN2^ΔC125G^ levels were pooled before further analysis.

### Preparation of conditioned media

MLF CMs were prepared from 90% confluent cultures by incubating the cells for 48 hours in serum-free DMEM. The MLF CM was passed through a 0.2 μm Millipore filter and either used immediately or stored at −70°C after shock-freezing in liquid nitrogen. For western blot analysis, the MLF CMs were concentrated by passing through a 3-kDa cut-off filtration membrane (Vivaspin 20, Sartorius).

For A549 CM, 1×10^5^ cells were seeded onto six-well plates and incubated for 24 hours in complete DMEM before exposure to MLF CMs for an additional 48 hours. Like the MLF CMs, the A549 CMs were passed through a 0.2 μm Millipore filter prior to use or storage.

For KGF and TGFβ neutralisation, anti-KGF or -pan-TGFβ antibodies were added to the conditioned media at a concentration of 10 μg/ml or 15 μg/ml, respectively.

### Histology, immunohistochemistry and immunofluorescence

Paraffin sections of lung tissue were prepared and stained using standard histology procedures. For immunostainings, 5-μm tissue sections were deparaffinised, rehydrated, boiled for 20 minutes in a microwave in citrate buffer, pH 6.0 (Invitrogen), and cooled down for 30 minutes. After rinsing in dH_2_O and PBS for 5 minutes the slides were treated for 10–15 minutes with H_2_O_2_ Block (Thermo Scientific) and for 5 minutes with Ultra V Block (Thermo Scientific) to inactivate the endogenous peroxidases. After rinsing in dH_2_O and soaking in PBS for 5–10 minutes, the slides were treated for 1 hour with 2% BSA in PBS to saturate nonspecific protein-binding sites. The slides were then exposed to the specific antibodies with 2% BSA in PBS at 4°C overnight. After removing excess antibody, the slides were treated with appropriate biotin-labelled secondary antibodies (KPL) at room temperature (RT) for 30 minutes, and finally with streptavidin peroxidase (KPL) at RT for 30 minutes. After washing, the slides were incubated in AEC Chromogen Single Solution (Thermo Scientific) at RT for 10–20 minutes. Finally, the slides were counterstained with Mayer’s Haematoxylin Solution (Sigma) and embedded in ImmunoHistoMount (Santa Cruz Biotechnology). For TTF1 immunostainings, the M.O.M. Kit (Mouse on Mouse Kit, Vector Laboratories) staining procedure was used according to the manufacturer’s instructions.

For tissue immunofluorescence, 5-μm thick, deparaffinised tissue sections were treated for 30 minutes with 2% BSA in PBS followed by an avidin/biotin block using the Endogenous Biotin-Blocking Kit (Invitrogen). After an overnight incubation with primary antibody at 4°C and removal of the excess antibody, the sections were treated with biotinylated secondary antibody (Kirkegaard and Perry Laboratories) at RT for 30 minutes and subsequently incubated for another 30 minutes with fluorochrome-conjugated streptavidin (streptavidin-Cy3, Biolegend/Biozol). The slides were mounted in DAPI containing Fluoroshield (Sigma-Aldrich).

For the analysis of membrane ruffling, MLFs were grown on coverslips, starved for 24 hours and then treated with 25 ng/ml PDGF-BB for the indicated time. Cells were fixed with 4% paraformaldehyde in PBS, blocked and permeabilised for 10 minutes with 1% BSA in PBS supplemented with 0.5% Triton X-100, labelled with primary antibody for 1 hour and then stained with Alexa-Flour-488-conjugated secondary antibody (Invitrogen) and Phalloidin 594 fluorescent dye (Invitrogen) for 45 minutes at RT. The cells were embedded in Mowiol mounting medium and the images were taken with a confocal laser-scanning microscope (Zeiss LSM510 Meta).

### Elastic fibre quantification by image analysis

To visualise elastic fibres, 3-μm paraffin sections were stained with Weigert’s resorcin-fuchsin solution and counterstained with nuclear fast red. Slides were analysed by light microscopy at 40× magnification using the Leica Qwin software. Depending on the size of the lung section, between 175 and 370 images were analysed. Intact elastin fibres were counted within an area of 0.07598346 mm^2^ for each image and the number of fibres was calculated according to the following equation:

$$\text{intact}\;\text{fibres}=\frac{\sum \text{intact}\;\text{fibres}}{\sum \text{images}\times 0.07598346\;{\text{mm}}^{2}}.$$

### *In situ* hybridisation coupled with immunofluorescence

The *Sesn2* riboprobe was prepared by PCR as previously described (Mittal et al., 2007) using 5′-AATTAACCCTCACTAAAGGGATAACACCATCGCCATGCAC-3′ and 5′-AATTAACCCTCACTAAAGGGATAACACCATCGCCATGCAC-3′ as *Sesn2*-specific primers. Hybridisation of the *Sesn2* antisense probe to *Sesn2* mRNA was visualised by Alexa Fluor 555 tyramide fluorescent substrate (Invitrogen, Karlsruhe, Germany). α-SMA was detected with an FITC-conjugated anti-α-SMA mouse monoclonal antibody (F3777, Sigma).

### ROS measurements

Superoxide release from MLFs was measured by EPR as described (Mittal et al., 2007). Briefly, EPR measurements were performed at −170°C using an EMXmicro Electron Spin Resonance (ESR) spectrometer (Bruker, Karlsruhe, Germany) using 1-hydroxy-3-methoxycarbonyl-2,2,5,5-tetramethylpyrrolidine (CMH, Noxygen) as the spin probe for detecting intra- and extracellular superoxide production. Because CMH reacts with superoxide and peroxynitrite, parallel samples containing either CMH alone or CMH and superoxide dismutase (SOD) conjugated to polyethyleneglycol (PEG-SOD) were measured. This enabled the assessment of the superoxide signal as part of the total CMH signal. Thus, duplicate samples of 2×10^5^ cells were incubated with 15 U/ml PEG-SOD (Sigma) for 2 hours at 37°C followed by the addition of CMH (500 μM) ± PEG-SOD. After incubating for another 20 minutes, the samples were shock-frozen and stored in liquid nitrogen. Spectrometry was performed on frozen samples using a g-factor of 2.0063, a centre field of 3349.95G, a microwave power of 200 mW, a sweep time of 20 seconds and a sweep number of 5.

### Nucleic acids and protein analyses

Total cellular RNA was isolated using TriReagent (Sigma) according to the manufacturer’s instructions. Real-time RT-PCR analysis of gene expression was performed using SYBR Green (ABgene, Epsom, UK) and/or TaqMan chemistry (Life Technologies) in an Opticon 2 qPCR machine (MJ Research). cDNA was synthesised from total RNA using random priming and Superscript II reverse transcriptase (Invitrogen). PCR reactions were run as triplicates on 96-well plates, with each reaction containing cDNA derived from 7.5–15 ng of total RNA, 1× ABsolute SYBR fluorescein mix (ABGene), and 5 pmol of gene-specific primers in a total volume of 25 μl. All gene-specific primers are available on request. Reactions were normalised by simultaneously carrying out RT-PCR reactions for RNApolII using the primers 5′-ATGAGCTGGAACGGGAATTTGA-3′ and 5′-ACCACTTTGATGGGATGCAGGT-3′. The temperature profile was 10 minutes at 94°C followed by 40 cycles at 94°C for 15 seconds, 61°C for 30 seconds, and 72°C for 30 seconds.

Western blotting was performed as previously described (Heidler et al., 2011) using the specific antibodies listed above.

### Patient characteristics

Human lung tissues were obtained from transplanted COPD transplant patients (GOLD stage IV), smokers without COPD, and donor controls. The studies were approved by the Ethics Committee of the Justus-Liebig-University School of Medicine (AZ 31/93), Giessen, Germany.

### Statistical analysis

Comparisons between more than two groups were performed using analysis of variance (ANOVA) with the Student-Newman-Keuls post test. For comparisons of two groups, Student’s two-tailed *t*-test was used. *P*-values below 0.05 were considered significant.

## Supplementary Material

Supplementary Material

## References

[b1-0061378] Bae S. H. Sung S. H. Oh S. Y. Lim J. M. Lee S. K. Park Y. N. Lee H. E. Kang D. Rhee S. G. (2013). Sestrins activate Nrf2 by promoting p62-dependent autophagic degradation of Keap1 and prevent oxidative liver damage. Cell Metab. 17, 73–84 23274085 10.1016/j.cmet.2012.12.002

[b2-0061378] Boström H. Willetts K. Pekny M. Levéen P. Lindahl P. Hedstrand H. Pekna M. Hellström M. Gebre-Medhin S. Schalling M. (1996). PDGF-A signaling is a critical event in lung alveolar myofibroblast development and alveogenesis. Cell 85, 863–873 8681381 10.1016/s0092-8674(00)81270-2

[b3-0061378] Budanov A. V. Karin M. (2008). p53 target genes sestrin1 and sestrin2 connect genotoxic stress and mTOR signaling. Cell 134, 451–460 18692468 10.1016/j.cell.2008.06.028 PMC2758522

[b4-0061378] Budanov A. V. Shoshani T. Faerman A. Zelin E. Kamer I. Kalinski H. Gorodin S. Fishman A. Chajut A. Einat P. (2002). Identification of a novel stress-responsive gene Hi95 involved in regulation of cell viability. Oncogene 21, 6017–6031 12203114 10.1038/sj.onc.1205877

[b5-0061378] Budanov A. V. Sablina A. A. Feinstein E. Koonin E. V. Chumakov P. M. (2004). Regeneration of peroxiredoxins by p53-regulated sestrins, homologs of bacterial AhpD. Science 304, 596–600 15105503 10.1126/science.1095569

[b6-0061378] Chedid M. Rubin J. S. Csaky K. G. Aaronson S. A. (1994). Regulation of keratinocyte growth factor gene expression by interleukin 1. J. Biol. Chem. 269, 10753–10757 7511604

[b7-0061378] Choi M. H. Lee I. K. Kim G. W. Kim B. U. Han Y. H. Yu D. Y. Park H. S. Kim K. Y. Lee J. S. Choi C. (2005). Regulation of PDGF signalling and vascular remodelling by peroxiredoxin II. Nature 435, 347–353 15902258 10.1038/nature03587

[b8-0061378] Dabovic B. Chen Y. Choi J. Vassallo M. Dietz H. C. Ramirez F. von Melchner H. Davis E. C. Rifkin D. B. (2009). Dual functions for LTBP in lung development: LTBP-4 independently modulates elastogenesis and TGF-beta activity. J. Cell. Physiol. 219, 14–22 19016471 10.1002/jcp.21643 PMC2719250

[b9-0061378] Dumitrascu R. Weissmann N. Ghofrani H. A. Dony E. Beuerlein K. Schmidt H. Stasch J. P. Gnoth M. J. Seeger W. Grimminger F. (2006). Activation of soluble guanylate cyclase reverses experimental pulmonary hypertension and vascular remodeling. Circulation 113, 286–295 16391154 10.1161/CIRCULATIONAHA.105.581405

[b10-0061378] Finkel T. (2011). Signal transduction by reactive oxygen species. J. Cell Biol. 194, 7–15 21746850 10.1083/jcb.201102095 PMC3135394

[b11-0061378] Fischer H. Taylor N. Allerstorfer S. Grusch M. Sonvilla G. Holzmann K. Setinek U. Elbling L. Cantonati H. Grasl-Kraupp B. (2008). Fibroblast growth factor receptor-mediated signals contribute to the malignant phenotype of non-small cell lung cancer cells: therapeutic implications and synergism with epidermal growth factor receptor inhibition. Mol. Cancer Ther. 7, 3408–3419 18852144 10.1158/1535-7163.MCT-08-0444 PMC2879863

[b12-0061378] Hägerstrand D. Hesselager G. Achterberg S. Wickenberg Bolin U. Kowanetz M. Kastemar M. Heldin C. H. Isaksson A. Nistér M. Ostman A. (2006). Characterization of an imatinib-sensitive subset of high-grade human glioma cultures. Oncogene 25, 4913–4922 16547494 10.1038/sj.onc.1209497

[b13-0061378] Heidler J. Al-Furoukh N. Kukat C. Salwig I. Ingelmann M. E. Seibel P. Krüger M. Holtz J. Wittig I. Braun T. (2011). Nitric oxide-associated protein 1 (NOA1) is necessary for oxygen-dependent regulation of mitochondrial respiratory complexes. J. Biol. Chem. 286, 32086–32093 21771794 10.1074/jbc.M111.221986 PMC3173199

[b14-0061378] Hersh C. P. Demeo D. L. Lazarus R. Celedón J. C. Raby B. A. Benditt J. O. Criner G. Make B. Martinez F. J. Scanlon P. D. (2006). Genetic association analysis of functional impairment in chronic obstructive pulmonary disease. Am. J. Respir. Crit. Care Med. 173, 977–984 16456143 10.1164/rccm.200509-1452OC PMC2662917

[b15-0061378] Kolla V. Gonzales L. W. Gonzales J. Wang P. Angampalli S. Feinstein S. I. Ballard P. L. (2007). Thyroid transcription factor in differentiating type II cells: regulation, isoforms, and target genes. Am. J. Respir. Cell Mol. Biol. 36, 213–225 16960125 10.1165/rcmb.2006-0207OC PMC1899316

[b16-0061378] Levéen P. Pekny M. Gebre-Medhin S. Swolin B. Larsson E. Betsholtz C. (1994). Mice deficient for PDGF B show renal, cardiovascular, and hematological abnormalities. Genes Dev. 8, 1875–1887 7958863 10.1101/gad.8.16.1875

[b17-0061378] Manning B. D. (2004). Balancing Akt with S6K: implications for both metabolic diseases and tumorigenesis. J. Cell Biol. 167, 399–403 15533996 10.1083/jcb.200408161 PMC2172491

[b18-0061378] Mellström K. Heldin C. H. Westermark B. (1988). Induction of circular membrane ruffling on human fibroblasts by platelet-derived growth factor. Exp. Cell Res. 177, 347–359 3391248 10.1016/0014-4827(88)90468-5

[b19-0061378] Mittal M. Roth M. König P. Hofmann S. Dony E. Goyal P. Selbitz A. C. Schermuly R. T. Ghofrani H. A. Kwapiszewska G. (2007). Hypoxia-dependent regulation of nonphagocytic NADPH oxidase subunit NOX4 in the pulmonary vasculature. Circ. Res. 101, 258–267 17585072 10.1161/CIRCRESAHA.107.148015

[b20-0061378] Ohnishi K. Sakai F. Kudoh S. Ohno R. (2006). Twenty-seven cases of drug-induced interstitial lung disease associated with imatinib mesylate. Leukemia 20, 1162–1164 16598305 10.1038/sj.leu.2404207

[b21-0061378] Rahman I. (2012). Pharmacological antioxidant strategies as therapeutic interventions for COPD. Biochim. Biophys. Acta 1822, 714–728 22101076 10.1016/j.bbadis.2011.11.004 PMC3295924

[b22-0061378] Rahman I. MacNee W. (2012). Antioxidant pharmacological therapies for COPD. Curr. Opin. Pharmacol. 12, 256–265 22349417 10.1016/j.coph.2012.01.015 PMC3768007

[b23-0061378] Repapi E. Sayers I. Wain L. V. Burton P. R. Johnson T. Obeidat M. Zhao J. H. Ramasamy A. Zhai G. Vitart V. Wellcome Trust Case Control Consortium NSHD Respiratory Study Team (2010). Genome-wide association study identifies five loci associated with lung function. Nat. Genet. 42, 36–44 20010834 10.1038/ng.501 PMC2862965

[b24-0061378] Seimetz M. Parajuli N. Pichl A. Veit F. Kwapiszewska G. Weisel F. C. Milger K. Egemnazarov B. Turowska A. Fuchs B. (2011). Inducible NOS inhibition reverses tobacco-smoke-induced emphysema and pulmonary hypertension in mice. Cell 147, 293–305 22000010 10.1016/j.cell.2011.08.035

[b25-0061378] Shapiro S. D. (2000). Animal models for chronic obstructive pulmonary disease: age of klotho and marlboro mice. Am. J. Respir. Cell Mol. Biol. 22, 4–7 10615058 10.1165/ajrcmb.22.1.f173

[b26-0061378] Shin B. Y. Jin S. H. Cho I. J. Ki S. H. (2012). Nrf2-ARE pathway regulates induction of Sestrin-2 expression. Free Radic. Biol. Med. 53, 834–841 22749810 10.1016/j.freeradbiomed.2012.06.026

[b27-0061378] Sterner-Kock A. Thorey I. S. Koli K. Wempe F. Otte J. Bangsow T. Kuhlmeier K. Kirchner T. Jin S. Keski-Oja J. (2002). Disruption of the gene encoding the latent transforming growth factor-beta binding protein 4 (LTBP-4) causes abnormal lung development, cardiomyopathy, and colorectal cancer. Genes Dev. 16, 2264–2273 12208849 10.1101/gad.229102 PMC186672

[b28-0061378] Thamsen M. Kumsta C. Li F. Jakob U. (2011). Is overoxidation of peroxiredoxin physiologically significant? Antioxid. Redox Signal. 14, 725–730 20964547 10.1089/ars.2010.3717 PMC3021361

[b29-0061378] Tuder R. M. Petrache I. (2012). Pathogenesis of chronic obstructive pulmonary disease. J. Clin. Invest. 122, 2749–2755 22850885 10.1172/JCI60324 PMC3408733

[b30-0061378] Ware L. B. Matthay M. A. (2002). Keratinocyte and hepatocyte growth factors in the lung: roles in lung development, inflammation, and repair. Am. J. Physiol. 282, L924–L940 10.1152/ajplung.00439.2001 11943656

[b31-0061378] Weichhart T. Costantino G. Poglitsch M. Rosner M. Zeyda M. Stuhlmeier K. M. Kolbe T. Stulnig T. M. Hörl W. H. Hengstschläger M. (2008). The TSC-mTOR signaling pathway regulates the innate inflammatory response. Immunity 29, 565–577 18848473 10.1016/j.immuni.2008.08.012

[b32-0061378] Wempe F. De-Zolt S. Koli K. Bangsow T. Parajuli N. Dumitrascu R. Sterner-Kock A. Weissmann N. Keski-Oja J. von Melchner H. (2010). Inactivation of sestrin 2 induces TGF-beta signaling and partially rescues pulmonary emphysema in a mouse model of COPD. Dis. Model. Mech. 3, 246–253 20106877 10.1242/dmm.004234

[b33-0061378] Woo H. A. Bae S. H. Park S. Rhee S. G. (2009). Sestrin 2 is not a reductase for cysteine sulfinic acid of peroxiredoxins. Antioxid. Redox Signal. 11, 739–745 19113821 10.1089/ars.2008.2360

[b34-0061378] Yildirim A. O. Muyal V. John G. Müller B. Seifart C. Kasper M. Fehrenbach H. (2010). Palifermin induces alveolar maintenance programs in emphysematous mice. Am. J. Respir. Crit. Care Med. 181, 705–717 20007933 10.1164/rccm.200804-573OC

[b35-0061378] Yoshida T. Tuder R. M. (2007). Pathobiology of cigarette smoke-induced chronic obstructive pulmonary disease. Physiol. Rev. 87, 1047–1082 17615396 10.1152/physrev.00048.2006

[b36-0061378] Yoshida T. Mett I. Bhunia A. K. Bowman J. Perez M. Zhang L. Gandjeva A. Zhen L. Chukwueke U. Mao T. (2010). Rtp801, a suppressor of mTOR signaling, is an essential mediator of cigarette smoke-induced pulmonary injury and emphysema. Nat. Med. 16, 767–773 20473305 10.1038/nm.2157 PMC3956129

