# Supplementary material for: Sestrin-2, a repressor of PDGFRβ signalling, promotes cigarette-smoke-induced pulmonary emphysema in mice and is upregulated in individuals with COPD
Source: Dis Model Mech. 2013 Aug 29;6(6):1378–87. doi: 10.1242/dmm.013482 (PMC3820261; doi:10.1242/dmm.013482)
Supplement: Supplementary Material [file supp_6_6_1378__index.html]

Sestrin-2, a repressor of PDGFRβ signalling, promotes cigarette-smoke-induced pulmonary emphysema in mice and is upregulated in individuals with COPD — Sestrin-2, a repressor of PDGFRβ signalling, promotes cigarette-smoke-induced pulmonary emphysema in mice and is upregulated in individuals with COPD — Supplementary Material 

# Sestrin-2, a repressor of PDGFRβ signalling, promotes cigarette-smoke-induced pulmonary emphysema in mice and is upregulated in individuals with COPD

## DMM013482 Supplementary Material

**Files in this Data Supplement:**

- **Supplementary Material PDF**
