# Supplementary material for: Sestrin-2, a repressor of PDGFRβ signalling, promotes cigarette-smoke-induced pulmonary emphysema in mice and is upregulated in individuals with COPD
Source: Dis Model Mech. 2013 Aug 29;6(6):1378–87. doi: 10.1242/dmm.013482 (PMC3820261; doi:10.1242/dmm.013482)
Supplement: Supplementary Material [file supp_013482_DMM013482.pdf]

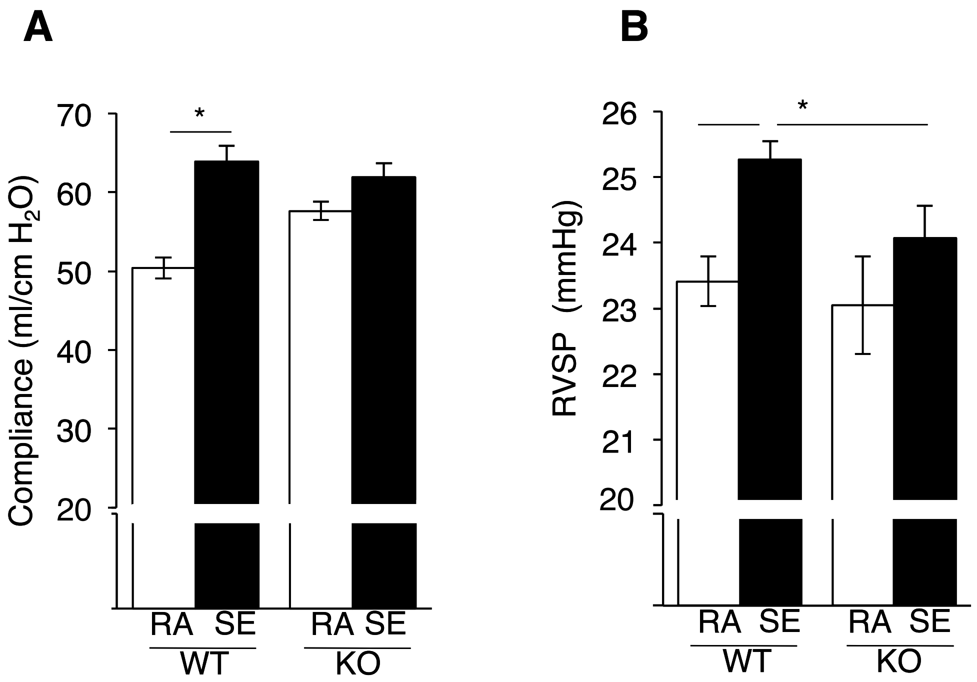

Fig. S1: Protection of *Sesn2* KO mice against cigarette smoke. **A.** Compliance. **B.** Right ventricular systolic blood pressure (n = 7-14 mice per group). RA, room air; SE, smoke exposed; WT, wild type; KO, *Sesn2* knockout. *$p < 0.05$.

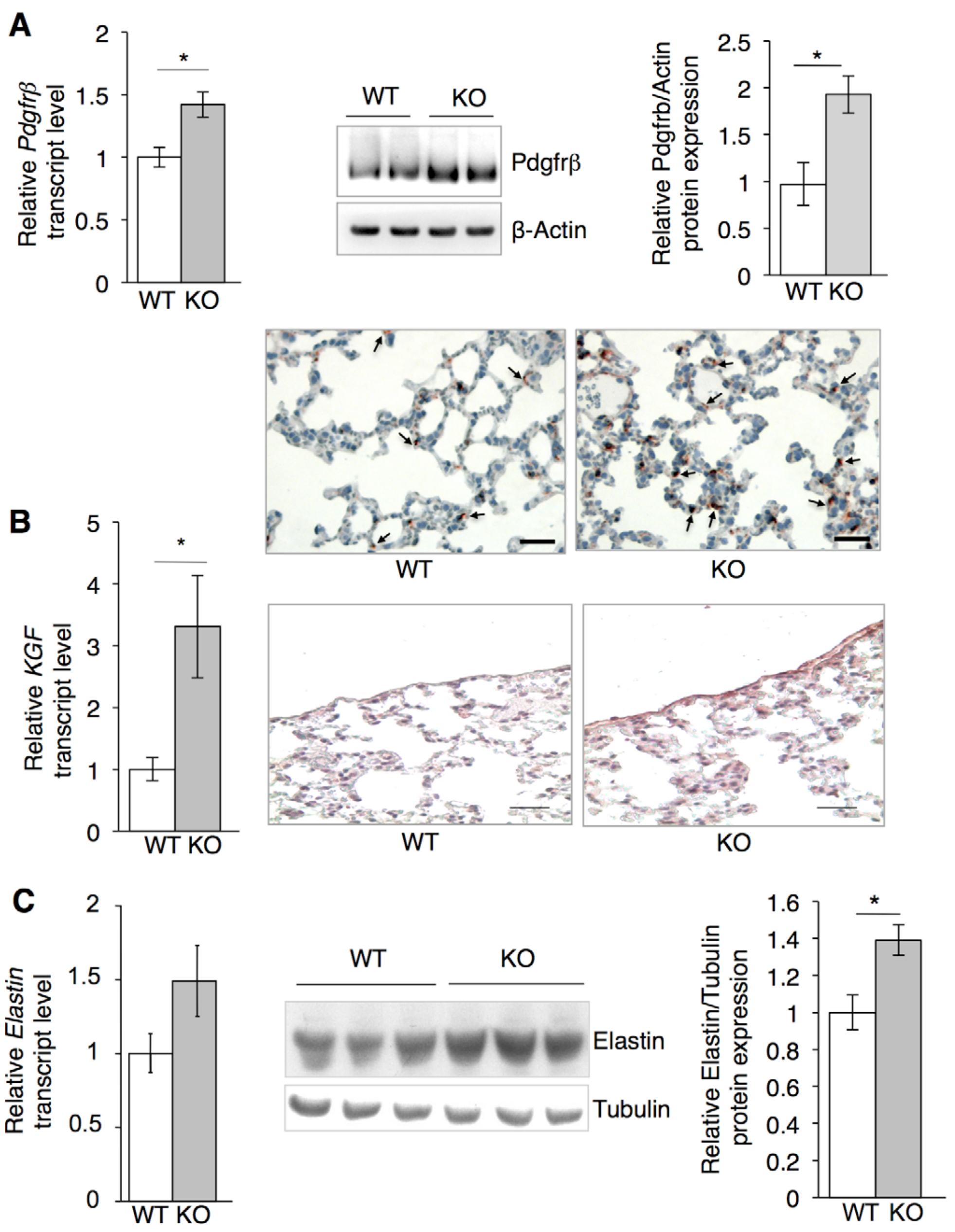

Fig. S2: Induction of alveolar maintenance programs in *Sesn2* KO mice *in vivo.* **A.** Upregulation of Pdgfrβ expression in *Sesn2* KO lungs. ***Upper panel***: (*Left*) *Pdgfrβ* mRNA in lung tissue homogenates quantified by qRT-PCR. (*Middle*) Representative Western blot probed with anti-PGDFRβ antibody. (*Right*) Densitometric quantification of Pdgfrβ expression. ***Lower panel:*** Representative lung sections stained with anti-PDGFRβ antibody and counterstained with haematoxilin. Pdgfrβ expressing cells (arrows) have brown stained cytoplasm. Scale bar: 50 μm. **B.** Upregulation KGF expression in *Sesn2* KO lungs. *Left panel:* KGF mRNA quantified by qRT-PCT in lung tissue homogenates. *Right panel:* Representative lung sections stained with anti-KGF antibody and counterstained with haematoxilin. Scale bar: 50 μm. **C.** Upregulation of elastin expression in *Sesn2* KO lungs. *Left panel: Elastin* mRNA quantified by qRT-PCR. *Middle panel:* Representative Western blot of total lung tissue homogenates. *Right panel:* Densitometric quantification of elastin expression. All results are represented as means ± SEM of lungs from n = 4-5 individual mice per group. *$p<0.05$.

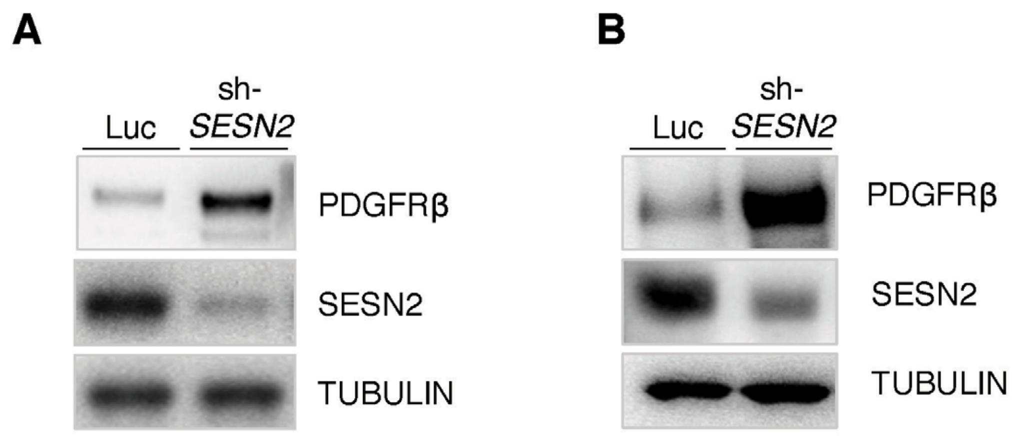

Fig. S3: Upregulation of PDGFRβ expression in MRC5 human fetal lung (**A**) and human adult primary lung fibroblasts (**B**) after lentiviral knockdown of *SESN2* shown by Western blotting using anti-PDGFRβ, anti-Sesn2 and anti-tubulin antibodies. *sh-SESN2*, *SESN2* shRNA*; Luc*, luciferase specific control shRNA.

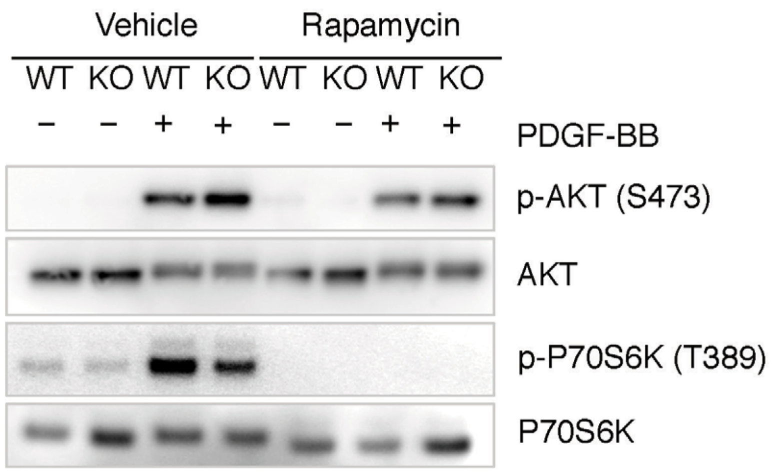

Fig. S4: Activation of mTORC1 signaling by PDGF-BB. Representative Western blot probed with anti-phospho-Akt (p-Akt), anti-Akt (Akt), anti-phospho-P70S6K (p-P70S6K), and anti-P70S6K (P706K) antibodies. Cell lysates prepared from serum-starved MLFs were exposed to 25 ng/ml recombinant human PDGF-BB for 20 min and to 100 nM rapamycin for 24 hours as indicated. Vehicle, DMSO.

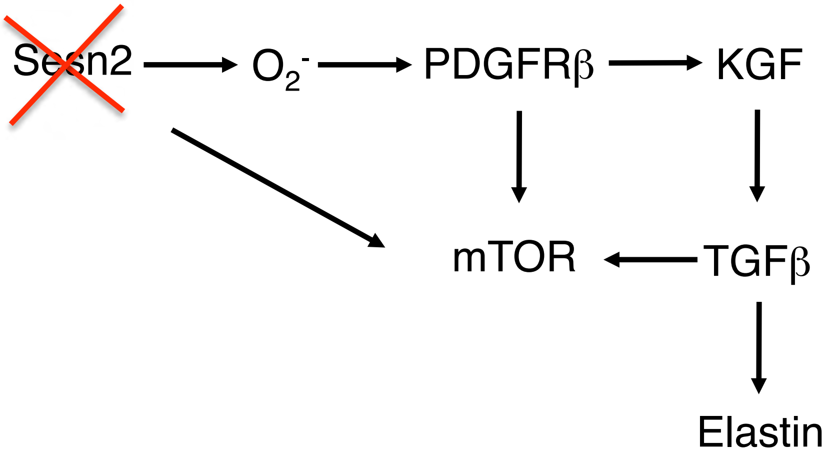

Fig S5: Schematic representation of signalling pathways affected by the Sesn2 mutation. Inactivation of Sesn2 derepresses mTOR and leads to an accumulation of superoxide anions (O_2^-) which upregulate PDGFRβ. Amplification of PDGF signalling induces KGF secretion by lung parenchymal fibroblasts and stimulates the mTOR even further. KGF promotes ATII cell proliferation and secretion of TGFβ to upregulate elastin. Alltogether, the activation of these pathways protect against pulmonary emphysema caused by cigarette smoke.

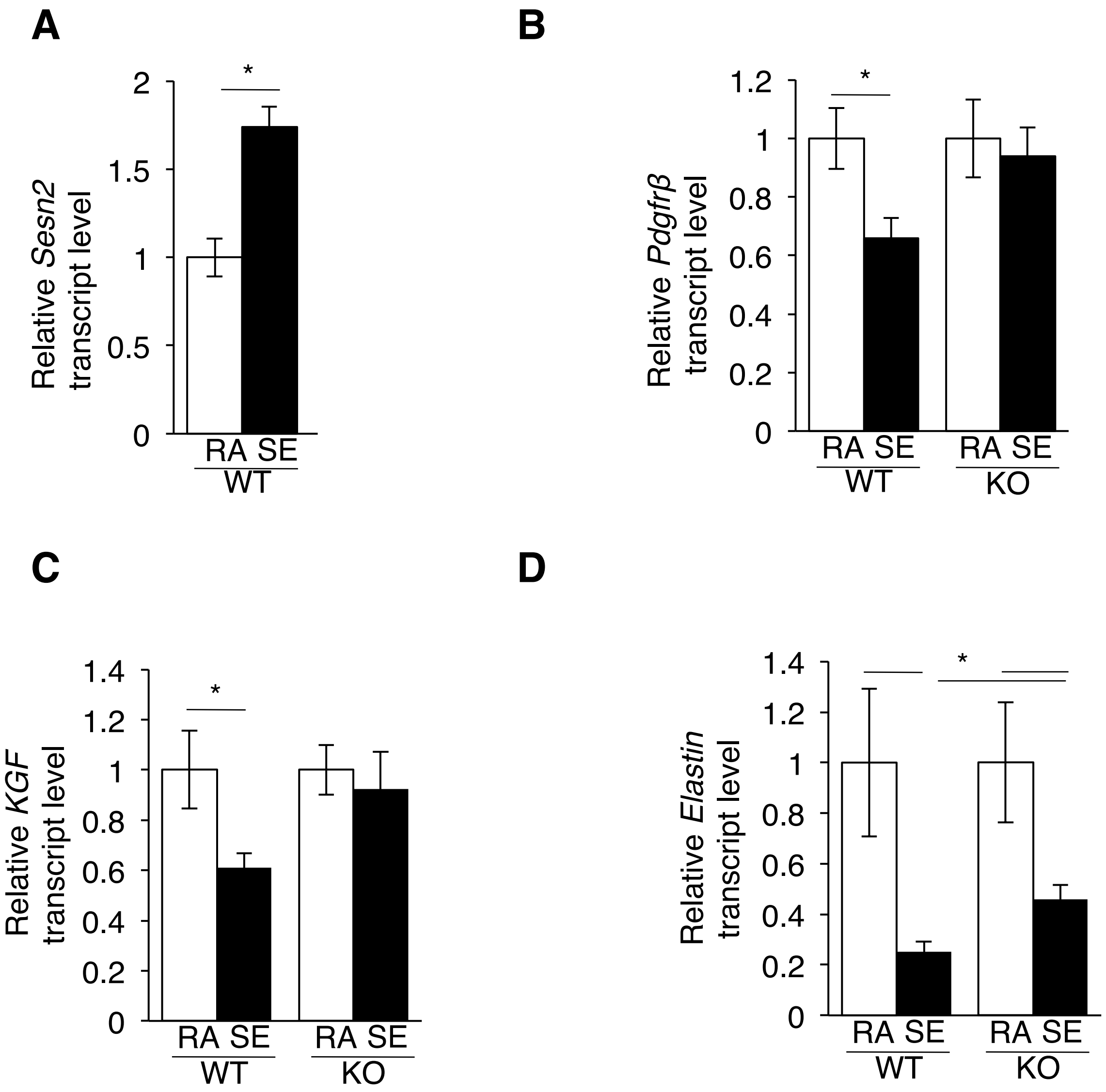

Fig. S6: Upregulation of *Sesn2* expression and repression of alveolar maintenance programs in smoke exposed mice. mRNA levels of *Sesn2* **(A)**, *Pdgfrβ* **(B)**, *KGF* **(C)** and *elastin* **(D)** quantified by qRT-PCR from lung tissue homogenates of WT- and *Sesn2* KO mice exposed to room air (RA) or cigarette smoke (SE). Results are represented as means ± SEM of lungs from n= 5-7 mice per group. *p<0.05.

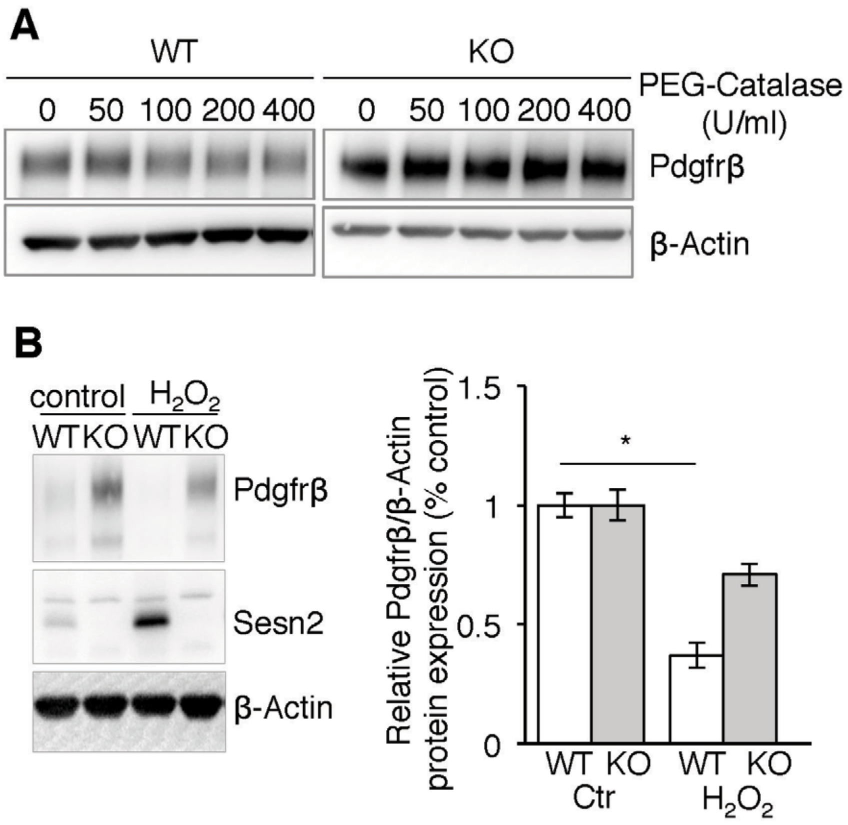

Fig. S7: ROS impact on PDGFRβ expression in MLFs. (**A**) Western blots showing Pdgfrβ expression in MLFs treated PEG-Catalase for 48 hours. **(B** and **C)** Pdgfrβ and Sesn2 expression in MLFs exposed for 6 hours to hydrogen peroxide (H_2O_2). **(B)** Representative Western blot. **(C)** Densitometric quantification of Pdgfrβ expression. Results are represented as means ± SEM of 3 separate expriments. *$p < 0.05$.

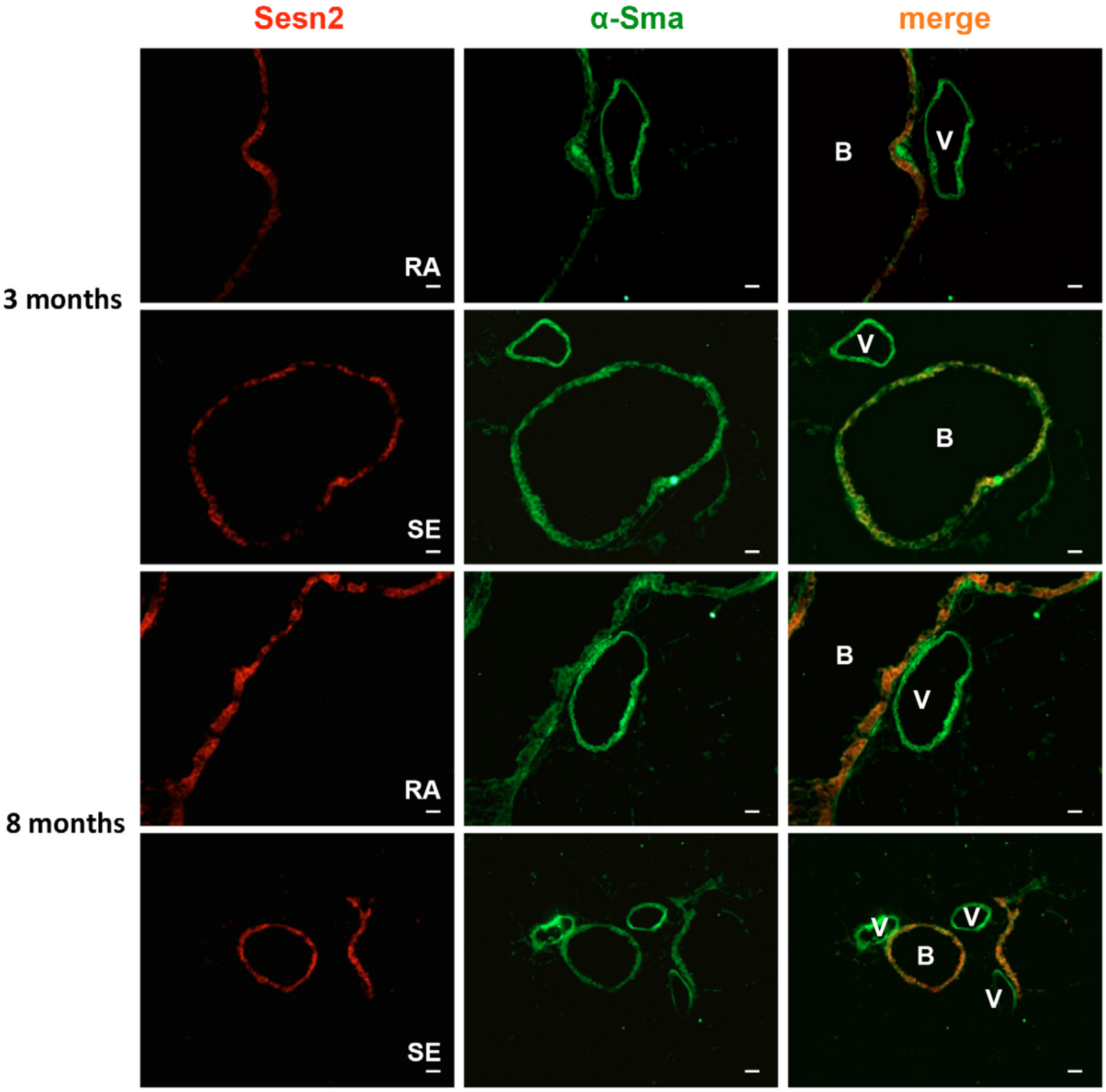

Fig. S8: Peribronchial *Sesn2* expression in lungs of wild type mice exposed to cigarette smoke. *Left panel: Sesn2* mRNA (red) visualized by fluorescence *in situ* hybridization. *Middle panel:* alpha-smooth muscle actin (α-Sma) (green) visualized by immunostaining. *Right panel:* Co-localization of *Sesn2* and α-Sma (*orange*) in subepithelial cells of the bronchial wall. Scale bar: 50 µm. RA, room air; SE, smoke-exposed.

Table S1: Clinical characteristics of normal donors, smokers, and COPD patients*

No.	Patient	Age	Sex	Pack/year	Diagnosis	FEV_1/FVC (%)	FEV_1(l)	FEV_1/predicted (%)
1	Donor	24	m	-	-	-	-	-
2	Donor	52	f	-	-	-	-	-
3	Donor	61	f	-	-	-	-	-
4	Donor	26	m	-	-	-	-	-
5	Donor	29	m	-	-	-	-	-
6	Donor	55	f	-	-	-	-	-
7	Donor	40	f	-	-	-	-	-
8	Donor	42	m	-	-	-	-	-
9	Donor	23	m	-	-	-	-	-
10	Donor	63	f	-	-	-	-	-
11	Donor	87	m	-	-	-	-	-
1	Smoker+COPD	53	m	39	COPD IV	49	1.56	14
2	Smoker+COPD	48	m	31	COPD IV	45	0.66	19
3	Smoker+COPD	58	m	70	COPD IV	31	0.86	20
4	Smoker+COPD	58	m	88	COPD IV	40	1.31	32
5	Smoker+COPD	59	m	5	COPD IV	63	0.76	22
6	Smoker+COPD	56	m	80	COPD IV	29	0.56	16
7	Smoker+COPD	55	m	35	COPD IV	38	0.55	13
8	Smoker+COPD	51	f	66	COPD IV	61	0.67	23
9	Smoker+COPD	63	m	37	COPD IV	40	0.93	20
10	Smoker+COPD	55	m	56	COPD IV	42	0.59	16
11	Smoker+COPD	50	f	60	COPD IV	41	0.57	23
12	Smoker+COPD	48	f	35	COPD IV	58	1.11	35
13	Smoker+COPD	45	m	60	COPD IV	34	1.00	20
1	Smoker w/o COPD	56	f	50	-	100	4.10	94
2	Smoker w/o COPD	87	m	10	-	71	3.20	63
3	Smoker w/o COPD	71	m	60	-	72	2.10	48
4	Smoker w/o COPD	60	f	10	-	68	3.80	79
5	Smoker w/o COPD	72	f	50	-	87	3.40	87

*COPD IV, Global Initiative for Chronic Obstructive Lung Disease (GOLD) stage IV; FEV1, forced expiratory volume per second; FVC, forced vital capacity.
